# A protocol for developing, disseminating, and implementing a core outcome set for stress urinary incontinence

**DOI:** 10.1097/MD.0000000000016876

**Published:** 2019-09-13

**Authors:** Maria-Patricia Rada, Vasilios Pergialiotis, Cornelia Betschart, Gabriele Falconi, Jorge Milhem Haddad, Stergios K. Doumouchtsis

**Affiliations:** a Department of Obstetrics and Gynaecology, Epsom & St Helier University Hospitals NHS Trust, London, United Kingdom; b “Iuliu Hatieganu” University of Medicine and Pharmacy Cluj-Napoca, 2nd Department of Obstetrics and Gynaecology, “Dominic Stanca” Clinic, Romania; c Laboratory of Experimental Surgery and Surgical Research N S Christeas, Athens University Medical School, Athens, Greece; d Department of Gynecology, University Hospital of Zurich, Zurich, Switzerland; e Department of Obstetrics and Gynaecology, San Bortolo Hospital, Vicenza, Italy; f Department Obstetrics and Gynaecology, Urogynaecology Division, Hospital das Clínicas da Faculdade de Medicina da Universidade de São Paulo, São Paulo, Brazil; g St George's University of London, London, UK.

**Keywords:** core outcome set, delphi survey, randomised controlled trials, stress urinary incontinence

## Abstract

**Introduction:**

Randomized trials evaluating interventions for stress urinary incontinence (SUI) have been using variable outcome measures, reporting a variety of outcomes. Alongside this variation across studies, outcome-reporting flaws contribute to a limited use of research to inform clinical practice. The development and use of core outcome sets (COSs) in future trials would ensure that outcomes important to different stakeholders and primarily women with SUI are reported more consistently and comprehensively.

**Methods:**

An international steering group including healthcare professionals, researchers, and women with urinary incontinence will guide the development of this COS. Potential outcomes will be identified through comprehensive literature reviews. These outcomes will be entered into an international, multiperspective online Delphi survey. All key stakeholders, including healthcare professionals, researchers, and women with urinary incontinence, will be invited to participate. The modified Delphi method encourages stakeholder group convergence toward collective agreement, also referred as consensus, core outcomes.

**Discussion:**

Dissemination and implementation of the resulting COS within an international context will be promoted and reviewed. Embedding the COS for SUI within future clinical trials, systematic reviews and clinical practice guidelines could make a significant contribution to advancing the value of research in informing clinical practice, enhancing patient care and improving outcomes. The infrastructure created by developing a COS for SUI could be leveraged in other settings, for example, selecting research priorities and clinical practice guideline development.

## Abstract 2

Supplemental Digital Content is available in the text

## 1 Introduction

Stress urinary incontinence (SUI) affects millions of women worldwide.^[1]^ Short- and long-term associated morbidity may have an effect on quality of life including daily activities, psychological wellbeing, and sexual function. The efficacy of the surgical and nonsurgical options to treat SUI has been studied in various clinical trials^[2–5]^; however, commonly and highly effective surgical treatments, such as the mid-urethral slings, are under scrutiny in the UK, Republic or Ireland, and elsewhere.

Current evidence has major flaws. In the absence of a standardized approach in research methodology, most SUI trials have reported information on various outcomes. For example, safety aspects of potential treatments for SUI, particularly over the longer term, were inconsistently evaluated across clinical trials. Even in the unlikely situation where outcomes have been consistently collected, evidence synthesis can be limited by the use of different outcome measures, including definitions and measurement instruments.

**Figure 1:**
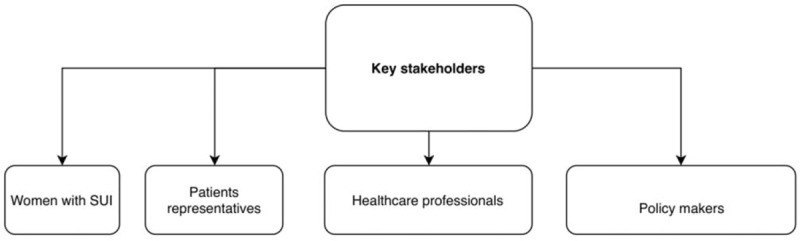
Key stakeholders involved in SUI. SUI = stress urinary incontinence.

Potential new treatments for SUI require an effective evaluation. The reporting of appropriate outcomes to reflect efficacy and safety of a treatment is a critical step in designing future randomized trials.^[6]^ To ensure relevance to policy and practice, the chosen outcomes need to be relevant to key stakeholders (Fig. 1).

The development and use of a collection of well-defined, discriminatory and feasible outcomes, termed a COS, would help to address these issues.^[7]^ COS are minimum datasets that can be measured in a standardized manner and reported consistently in the final publication.

**Figure 2:**
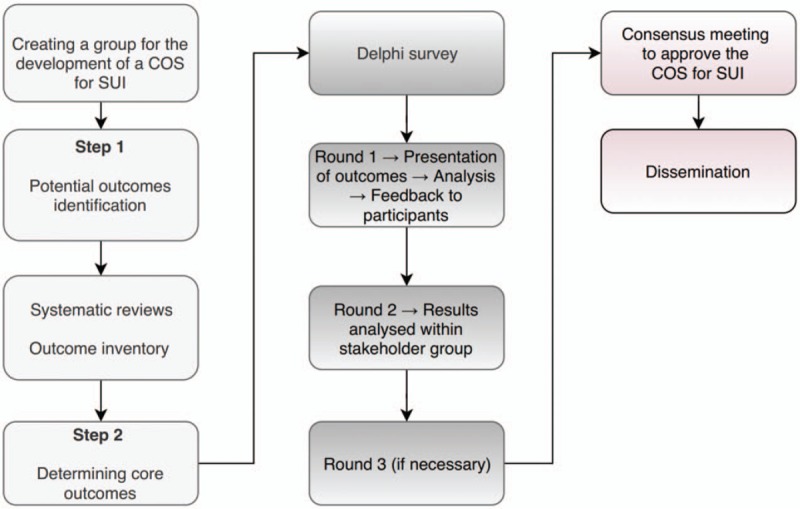
The steps of developing a COS for SUI. COS = core outcome set; SUI = stress urinary incontinence.

Our aim is to produce, disseminate, and implement a COS for SUI according to the Core Outcome Measures in Effectiveness Trials (COMET) guidelines,^[8]^ following the pathway illustrated by Figure 2.

## 2 Methods

### 2.1 Prospective registration

This study has been prospectively registered with the COMET initiative (registration number: 981) and is available online (www.comet-initiative.org/studies/details/981). This project is led and conducted by CHORUS, An International Collaboration for Harmonising Outcomes, Research, and Standards in Urogynaecology and Women's Health (https://i-chorus.org/).

### 2.2 Creating an international group for the development of a COS for SUI

An international steering group including healthcare professionals, researchers and women with SUI will guide the development of a COS that will be applicable to clinical studies evaluating therapeutic interventions for women with SUI. The development of effective interventions to reduce the effect of SUI on women's quality of life is urgently required, given the flaws and weaknesses documented in Cochrane reviews.^[6–7,9]^ Also, the scrutiny that previously considered “criterion standard” procedures such as the mid-urethral slings are currently undergoing by government bodies in the UK and Republic of Ireland resulted in a current high vigilance regime of restricted practice.^[10–11]^

### 2.3 Step 1: identifying potential outcomes

Selection of appropriate outcomes is an essential step for study design, as ultimately any study is only as valuable as its endpoints.^[8]^ Clinical trials that evaluate benefits and harms of interventions for SUI must select outcomes of relevance to key stakeholders and measure them using adequate tools. The main issues that arise throughout this process are inconsistent selection, measurement, and reporting of outcomes. A significant challenge is represented by the measurement of outcomes of interventions for SUI in a variety of ways that finally leads to outcome reporting bias. Therefore, the barrier to compare and highlight differences in clinical trials’ findings has an inevitable negative effect on their interpretation and embedding in clinical practice.

One way to address these issues is by going through a multi-step pathway (Fig. 2) that has been successfully applied in several disciplines, to develop, disseminate, and implement COS for various conditions. This protocol is in line with the COMET Initiative guidelines and other COS development research relevant to women's health including preeclampsia, endometriosis, pelvic organ prolapse, among others.^[7,12–14]^

For the development of this protocol and to evaluate the extent of existing variations we are currently completing a systematic review on the variation of outcomes and outcome measures in SUI randomised trials and we will generate an inventory of outcomes, outcome measures and definitions of both to inform the consensus process. The results will be entered into a modified Delphi method. Through the review we also aim to increase the range of evidence synthesis in SUI interventions field and to guide further research conduct towards the development of a COS.

#### 2.3.1 Systematic review (previously reported outcomes)

We identified trials evaluating therapeutic interventions for SUI following a standardized methodology and searches of databases including PubMed/ Medline, EMBASE, and Cochrane Central Register of Controlled Trials. We will evaluate publications that aim to improve the health of women with SUI, including trials and guidelines on various treatments including surgical interventions, physical therapies, and behavioral approaches.

#### 2.3.2 Outcome inventory

A comprehensive inventory of outcomes identified by systematic reviews will be developed. Outcome domains will be listed in a database and coded according to the taxonomy proposed by the COMET Initiative.

If there is uncertainty as to how to classify or present an outcome, consensus of the steering group will be sought. Following the steering group's agreement, the outcome inventory will be entered into the modified Delphi method.

### 2.4 Step 2: determining core outcomes

Delphi method involves a series of supervised rounds of surveys. The modified Delphi method encourages repeated reflection and rescoring of outcomes.^[15]^ These actions promote whole and individual stakeholder group convergence upon a consensus of core outcomes and has advantages over other agreement methods less standardized. Web-based Delphi surveys facilitate international participation and are considered feasible and efficient.^[16]^

Key stakeholders will be invited to participate in the Delphi survey. Stakeholders’ selection is meant to ensure inclusion of all relevant parties with an interest in SUI interventions. As there are no clear recommendations that we are aware of for the optimum sample size^[16]^ for a Delphi survey, based upon previous studies, we will aim to include 20 participants from each stakeholder group to ensure adequate representation.

The results of round 1 responder participants in each stakeholder group will be assessed at the end of this round. They will be presented as total number and/or percentage of:

- registrations
- respondents who have completed the survey
- respondents who completed the round
- respondents in each stakeholder group
- respondents compared to potential respondents as identified from the information provided by clinical leads
- new respondents who were not included in original invitation to complete the survey.

#### 2.4.1 Round 1

Participants will be invited to register online, provide demographic details, and commit to all rounds.^[17]^ They will be allocated a unique identifier which will make their responses anonymous. Individual outcomes will be scored by participants using a 7-point Likert Scale anchored between 1 (not important) and 7 (critical). This scale has been widely adopted by COS developers.^[18]^ During the first round, participants will be invited to suggest additional outcomes. Instead of limiting the variety of outcomes measured and reported across studies, a core outcome set would establish minimal reporting requirements while considering individual aspects. Additional outcomes noted by participants will be reviewed by the outcome committee and, if novel, listed in round 2. The round will close following a 4-week window.

#### 2.4.2 Round 2

Participants will present their individual and stakeholder group responses and they will be invited to reflect on the observed similarities and differences before proceeding to the next step and scoring individual outcomes again. The round will close following a 4-week window.

For each outcome, the median and H-spread of scores will be summarized graphically by stakeholder group and individual responses. Rescoring outcomes as part of the modified Delphi method facilitates agreement upon core outcomes.^[19]^ The round's 2 results will be reviewed and analyzed by the steering group to consider the need for a further Delphi survey round.

This round's results, as well as the results of any potential further round, will enable individual outcomes to be classified as shown in Table 1.^[16]^ Although subjective, these definitions and criteria have been proposed by previous COS developers,^[16,20–21]^ and help collecting and reporting uniform results.

**Table 1:**
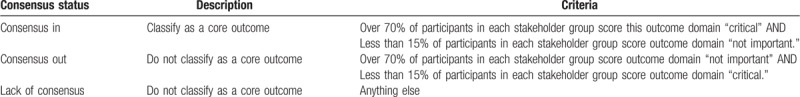
Consensus status based on core outcome criteria.

### 2.5 Stakeholder consultation

This final phase will involve a face-to-face meeting with key stakeholders and viewpoints from participants who have completed all steps of the survey. The objective of the consensus meeting will be to discuss consensus outcomes and approve a final COS for SUI interventions. During this meeting, the results from each round of the Delphi survey will be presented. To avoid biased consensus formation among group participants, the steering committee will ensure that the meeting is interactive and considers all opinions. To facilitate dissemination and implementation, professional society representatives, editors from key journals, and funders of SUI research^[22–23]^ will be invited to take part in this meeting.

## 3 Discussion

As with previous COS development projects, this project is not directly influencing patient safety,^[12,24–25]^ and therefore, ethics approval was not required. Also, The Medical Research Council decision tool that helps to determine whether a research project conducted within the UK requires approval from an NHS Research Ethics Committee (REC) indicated that this research does not require REC approval.

All involved participants will be asked for their consent (Supplemental Digital Content (Appendix 1)) before participating in either stakeholder meetings or the Delphi survey and all procedures will be conducted according to the Declaration of Helsinki.^[25]^ A “no-response” option will be allowed both for the survey and interactive parts of the research to ensure responder‘s right to withhold information. A specific timeframe of the Delphi process will be provided and information concerning the interval of data storage and handling will be made available to participants.

This COS development protocol will follow COS–Standards for Reporting (COS-STAR) statement and checklist.^[26]^ As dissemination is the crucial step in the effective application of trial outcomes, awareness must be increased among all involved parties to ensure widespread use of this COS in SUI interventions research and proper reporting.

Implementing COS in future clinical studies, systematic reviews and clinical guidelines could make a profound contribution to advancing the reach and relevance of research in informing clinical practice, enhancing patient care and improving intervention outcomes for women suffering from SUI. Mapping all outcomes reported in clinical trials assessing interventions for SUI in women will provide basis for offering best patient care for these patients.

### 3.1 Improving clinical trial outcome selection

Standard Protocol Items-Recommendations for Interventional Trials (SPIRIT) supported by funders of health research, recommend the use of COS where they exist.^[27]^ This study protocol was designed to the SPIRIT checklist.^[27]^ A COS that would hopefully ensure that consensus outcomes important to all stakeholders, including women with SUI are properly collected and reported. When clinical studies use consensus outcomes and outcome measures, meta-analyses, using individual patient data are feasible.

### 3.2 Improving clinical trial reporting and evidence synthesis

The Core Outcomes in Women's Health (CROWN) initiative, supported by several journals, have already implemented COS.^[23]^ Participating journals will require authors to report the results for core outcomes within trial reports and systematic reviews and offer conclusions based on these outcomes rather than non-core or surrogate outcomes. Where COSs have not been collected, the authors will be asked to report this limitation and its effect for their findings.^[22]^

### 3.3 Improving clinical practice guidelines

The National Institute for Health and Care Excellence (NICE) supports the use of COS when selecting outcomes during evidence scoping and synthesis.^[8]^ As this activity forms the basis of updating guideline recommendations, the COS could have a direct effecr in improving clinical practice.

## Acknowledgments

CHORUS, an International Collaboration for Harmonising Outcomes, Research and Standards in Urogynaecology and Women's Health (i-chorus.org).

## Author contributions

**Conceptualization:** Maria Rada, Vasilios Pergialiotis, Cornelia Betschart, Gabriele Falconi, Jorge Milhem Haddad, Stergios Doumouchtsis.

**Funding acquisition:** Stergios Doumouchtsis.

**Methodology:** Maria Rada.

**Project administration:** Stergios Doumouchtsis.

**Resources:** Maria Rada, Vasilios Pergialiotis, Cornelia Betschart, Jorge Milhem Haddad, Stergios Doumouchtsis.

**Supervision:** Stergios Doumouchtsis.

**Writing – original draft:** Maria Rada, Stergios Doumouchtsis.

**Writing – review & editing:** Maria Rada, Vasilios Pergialiotis, Cornelia Betschart, Gabriele Falconi, Jorge Milhem Haddad, Stergios Doumouchtsis.

## Supplementary Material

Supplemental Digital Content
