# Supplementary material for: A protocol for developing, disseminating, and implementing a core outcome set for stress urinary incontinence
Source: Medicine (Baltimore). 2019 Sep 13;98(37):e16876. doi: 10.1097/MD.0000000000016876 (PMC6750288; doi:10.1097/MD.0000000000016876)
Supplement: Supplemental Digital Content [file medi-98-e16876-s001.docx]

**Appendix 1**

**Delphi Survey consent form**

**A protocol for developing, disseminating, and implementing a Core Outcome Set for Stress Urinary Incontinence**

**Name of lead researcher**: Dr Maria-Patricia Rada

**Name of Delphi survey participant**: ………………………………………………………………………………………………………….

1. I confirm that I have read and understand the information sheet explaining the above research project and I have had the opportunity to ask questions about the project.
2. I understand that my participation is voluntary and that I am free to withdraw at any time without giving any reason and without there being any negative consequences. In addition, should I not wish to answer any particular question or questions, I am free to decline.
3. I give permission for my anonymised responses to be used during the Delphi process, and to be accessed by members of the research team. I understand that my name will not be linked with the research materials, and I will not be identifiable during the Delphi survey or in the reports that result from the research.
4. I agree to take part in the above research project.

**Date**:

**Participant's signature:**

**Completion**: Please return scanned or electronically completed forms via email to: [maria.rada@nhs.net](mailto:maria.rada@nhs.net). Alternatively please return hard copies by post to the following address: Dr Maria-Patricia Rada or Professor Stergios Doumouchtsis, Dorking Rd, Epsom KT18 7EG, Epsom General Hospital, K Block, Rowen House, Gynaecology Department.

**Copies**: Please retain a copy of the completed consent from for your personal records. An additional

copy will be held in a Hospital secure location for the duration of the research study.
